# Effects of a perpendicular ultrasonic field on planar and porous electrodes for hydrogen production in alkaline conditions

**DOI:** 10.1016/j.ultsonch.2025.107481

**Published:** 2025-07-27

**Authors:** Jérémy Gravelle, Vanessa Avramovic, Loïc Hallez, Jean-Yves Hihn, Bruno G. Pollet

**Affiliations:** a Green Hydrogen Lab (GH2Lab), Hydrogen Research Institute (HRI), Université du Québec à Trois-Rivières (UQTR), 3351 Boulevard des Forges, Trois-Rivières, Québec G9A 5H7, Canada; b Université Marie et Louis Pasteur, CNRS UMR 6213 Institut UTINAM, 30 Avenue de l’observatoire, 25009 Besançon Cedex, France

**Keywords:** Sonoelectrochemistry, Alkaline water electrolyser, Nickel, Hydrogen evolution reaction, Mass-transfer, Tafel, Dynamic hydrogen bubble template

## Abstract

Among water electrolysis systems, alkaline water electrolysers (AWE) are promising for large-scale hydrogen production due to their cost-effective nickel (Ni) electrodes. However, AWE faces challenges, particularly molecular hydrogen and oxygen bubbles shielding on electrode surfaces in the liquid alkaline electrolyte (KOH). Therefore, innovative solutions like ultrasonication and advanced electrode structures are explored as effective methods for bubble removal. This study investigates the hydrogen evolution reaction (HER) under two ultrasonic frequencies (20 kHz and 580 kHz) using planar and porous Ni electrodes on a copper (Cu) substrate. The planar electrode was prepared using a Ni watt bath, while the porous electrode was fabricated using the dynamic hydrogen bubble template (DHBT) method. To further align with industrial conditions, the distance between the electrodes and transducer was maintained between 10 and 15 cm. The study focused on the synergistic effect of ultrasonication and porous electrodes on mass transfer and HER performance. Results show that the porous Ni electrode reduced overpotential by −97 mV, with ultrasonication enhancing this by up to −132 mV. Furthermore, ultrasonication altered the HER mechanism for DHBT Ni, as shown by changes in the Tafel slopes, *b* (−250 mV/dec vs. −87 mV/dec under ultrasonication and silent conditions respectively). It was also found that the intense convection from acoustic cavitation implosion might influence HER mechanisms by hindering water adsorption at the electrode surface. Finally, the *electrode-sonotrode* distance was found to be critical to prevent surface degradation at shorter distances (3.5 cm), requiring further optimization for industrial purposes.

## 1 Introduction

In 2023, 97 Mt (million tonnes) of hydrogen was produced from fossil fuels worldwide leading to circa 920 Mt of CO_2_ released into the atmosphere [1]. This represents over 99 % of the total of hydrogen produced against less than 1 % produced from non-carbonaceous fuels. Among the low-emission hydrogen technologies, alkaline water electrolysis (AWE) accounts for 60 % of global hydrogen production through water electrolysis and is considered the most viable for worldwide implementation [1]. Indeed, investments and operating costs of these electrolyser systems are known to be fairly low compared to other water electrolysis methods, and AWE uses less expensive electrocatalysts such as nickel instead of more expensive options like platinum (Pt) and iridium (Ir) for PEM water electrolyser [2,3].

AWE splits water into molecular hydrogen (H_2_) and molecular oxygen (O_2_) by transferring hydroxide ions (OH^–^) between electrodes with the help of a separator or diaphragm. The respective reactions are called hydrogen evolution reaction (HER) (1) and oxygen evolution reaction (OER) (2).

(1) $$\text{Anode (OER)}\text{4O}{\text{H}}_{\left(\text{aq}\right)}^{-}\;\to \;{{\text{O}}_{\text{2}}}_{\left(\text{g}\right)}\;\text{+ 2}{\text{H}}_{\text{2}}{\text{O}}_{\text{(l)}}\;\text{+ 4}{\text{e}}^{\text{-}}{\text{E}}_{\text{O2/OH-}}^{\text{o}}\;=\;\text{+0.401}\;{\text{V}}_{\text{/SHE}}$$

(2) $$\text{Cathode (HER)}\text{2}{\text{H}}_{\text{2}}{\text{O}}_{\left(\text{l}\right)}\;\text{+ 2}{\text{e}}^{\text{-}}\;\to {{\;\text{H}}_{\text{2}}}_{\left(\text{g}\right)}\;\text{+ 2}{\text{OH}}_{\text{(aq)}}^{\text{-}}{\text{E}}_{\text{H2O/H2}}^{\text{o}}\;=\;-\text{0.828}\;{\text{V}}_{\text{/SHE}}$$

(3) $$\text{Overall reaction}\text{2}{\text{H}}_{\text{2}}{\text{O}}_{\text{(l)}}\to \;\text{2}{\text{H}}_{{\text{2}}_{\text{(g)}}}+{\text{O}}_{{\text{2}}_{\text{(g)}}}{\text{E}}_{R}^{\text{o}}\;=\;\text{1.229 V}$$

In alkaline media, the HER (2) is a multistep electrochemical process occurring at the electrode surface, which is known to proceed through a combination of three steps: (i) Volmer, (ii) Heyrovsky, and (iii) Tafel steps, each presented in Table 1 and Table 2 with their respective theoretical Tafel slope values. The three possible pathways are Volmer-Heyrovsky, Volmer-Tafel, and Volmer-Heyrovsky-Tafel, with their Tafel slopes typically around 116–120 mV.dec^−1^ for nickel electrodes in alkaline electrolyte solutions, as the Volmer step is generally the rate-determining step (rds). However, lower *b* values (<100 mV.dec^−1^) and higher *b* values (>140 mV.dec^−1^) are often observed for Ni electrodes possessing non-planar shapes and extended surface areas or significant porosity, such as porous Ni and Raney Ni [[4], [5], [6]].

**Table 1:** HER mechanisms in alkaline media [4,6].

Mechanism	Rate-determining step (rds)	Tafel slope (mV.dec^−1^)
Volmer – Heyrovsky	${\text{H}}_{\text{2}}\text{O}\;+{\;\text{e}}^{\text{-}}\;\to \;{\text{H}}_{\text{ads}}\;+\;\text{O}{\text{H}}^{\text{-}}$	120
	Volmer	
Volmer – Heyrovsky	${\text{H}}_{\text{ads}}\;+{\;\text{H}}_{\text{2}}\text{O}\;+{\;\text{e}}^{\text{-}}\;\to \;{\;\text{H}}_{\text{2}}\;+\;\text{O}{\text{H}}^{\text{-}}$	40
	Heyrovsky	
Volmer – Tafel	${\text{2 H}}_{\text{2}}\text{O}\;+{\;\text{2}\text{e}}^{\text{-}}\;\to \;\text{2}{\text{H}}_{\text{ads}}\;+\;\text{2}\text{O}{\text{H}}^{\text{-}}$	60
	Volmer	
Volmer – Tafel	$\text{2}{\text{H}}_{\text{ads}}\;\to \;{\;\text{H}}_{\text{2}}$	30
	Tafel

**Table 2:** Roughness parameters of both planar and porous nickel electrodeposited electrode.

Parameters	Planar	DHBT
*S*_a_ (μm)	0.25	5.27
*S*_q_ (μm)	0.35	7.04
*S*_ku_	6.93	4.82
*S*_sk_	0.63	−1.47
*β_x_*	2.8	2.2
*β_y_*	2.2	2.1

Furthermore, the reaction (3) starts with the standard equilibrium voltage of 1.229 V (at 298.15 K, 1 atm, and pH 14 [7]), representing the baseline for water electrolysis. To achieve practical reaction rates, overpotentials must be added to *U*_eq_, including activation overpotentials (*η*_act_) and ohmic losses (*RI*). These ohmic losses stem from the electrolyte resistance (*R*_e_), membrane resistance (*R*_ms_), connection resistance (*R*_c_), and bubble resistance (*R*_b_), formed by gas formation at low temperatures [[8], [9], [10], [11]].

(4) $${\text{U}}_{\text{cell}}={\text{U}}_{\mathrm{eq}}\;+\eta_{\text{a}\text{ct}}\;\text{+ (}{\text{R}}_{\text{e}}+{\;\text{R}}_{\text{ms}}+{\text{R}}_{\text{c}}+{\text{R}}_{\text{b}}\text{).I}\;$$

Molecular hydrogen and oxygen bubble formation are one of the primary power losses of an AWE [12] by covering the electrode surface areas and hence blocking further electrochemical reduction/oxidation, creating thermal inhomogeneities [12,13], and modifying the local environment of the electrolyte [14]. To reduce overpotentials and bubble resistance, several strategies have been developed, which can be broadly categorized into two main approaches [15]: (i) passive strategies, which involve modifying system components to enhance properties and (ii) active strategies, which incorporate external energy inputs to improve performance.

Numerous studies have been conducted to enhance the surface properties of nickel-based electrocatalysts in passive strategies, employing the following main types of processes: (i) by increasing the gas surface tension, called *superaerophobicity* by the authors [16], (ii) by depositing active materials onto *pre*-roughened or porous surfaces through methods such as electrodeposition [[17], [18], [19]], or by using dynamic hydrogen bubble template (DHBT) [20], hydrothermal [21] or multi-step deposition [22], and other additive manufacturing methods [[23], [24], [25]], (iii) by using destructive processes like laser structuring [26,27], ultrasonic structuring [28], forcing a corrosion process [29] which directly creates the desired surface characteristics on the electrocatalyst surface, (iv) by using hybrid processes, which is composed of an additive step then a destructive step, like the electroplating of Raney nickel-alloy [30,31] or the sputtering with heat treatment [32] then leaching the undesired elements, creating porosities and rough structures.

Active strategies to reduce bubble resistance primarily include the use of: mechanical stirring [11], magnetic fields [33,34], supergravity fields [35], and power ultrasound [6,36]. The latter is particularly noteworthy because it leverages *Bjerknes forces* to accelerate bubble coalescence and ejection [[37], [38], [39], [40]]. *Bjerknes forces* are made of two components: (a) *the primary Bjerknes force*, which governs the movement of individual bubbles according to their size relative to their resonance frequency, where smaller bubbles are attracted toward regions of higher pressure and larger ones are ejected [41], and (b) the *secondary Bjerknes force*, which results from changes in the surrounding pressure gradient when bubbles are in close proximity, leading to mutual attraction and eventual coalescence of the small bubbles [42].

Additionally, ultrasound offers several other benefits [43] such as solution degassing, enhanced mass transport, electrode surface cleaning and activation, and disruption of the diffusion layer. With all these benefits, ultrasonication holds considerable potential as a process enhancement strategy for the sonoelectrocatalytic water-splitting reaction.

Foroughi *et al.* have extensively explored the role of ultrasonication in enhancing HER and OER enhancement in both acidic [44] and alkaline [6,36,45] environments. Their findings underscore the promising application of ultrasonication as a *pre*-treatment technique, as it facilitates the formation of an active oxide layer [6], or as an auxiliary during electrolysis to mitigate bubble formation at the electrode surface [45]. Additional studies have investigated the integration of ultrasonic devices directly into lab-scale electrolysers to evaluate the effects of bubble removal *via* either direct [46] or indirect [47] ultrasonication.

The aim of the present study is to combine passive and active strategies by studying the effects of ultrasound on an electrode presenting a high roughness level i.e. an increased developed surface. This can be achieved by elaborating a DHBT nickel layer, and by comparing its performances to planar nickel coatings. A particular attention is paid to the localisation of the sample into the sonoelectrochemical reactor, and especially to the electrode-to-horn distances. Indeed, the distribution of the acoustic activity is inhomogeneous [48], because resonant systems distribute the acoustic energy in the ultrasonic horn axis. This results in a distribution of the “active zones” with cavitation activity localized into pressure nodes separated by half a wavelength (*λ*/2), i.e. every 37.5 mm for 20 kHz and 1.29 mm for 580 kHz in water. But this repartition is strongly influenced by the presence of obstacles [[49], [50], [51]], and it is highly recommended to use the electrodiffusionnal method [52,53] to measure the effective activity on the substrate surface. Therefore, mass transfer coefficient is used as a tool for local cavitation activity measurement at the substrate by electrodiffusionnal method [53], and water electrolysis is evaluated at the very same location, allowing to link the hydrogen generation efficiency to the effective agitation. This relationship has been successfully experimented in former experiments for coatings elaboration in copper electroless conditions [54] or silver electrodeposition [55].

## 2 Experimental procedures and methods

**Fig. 1:**
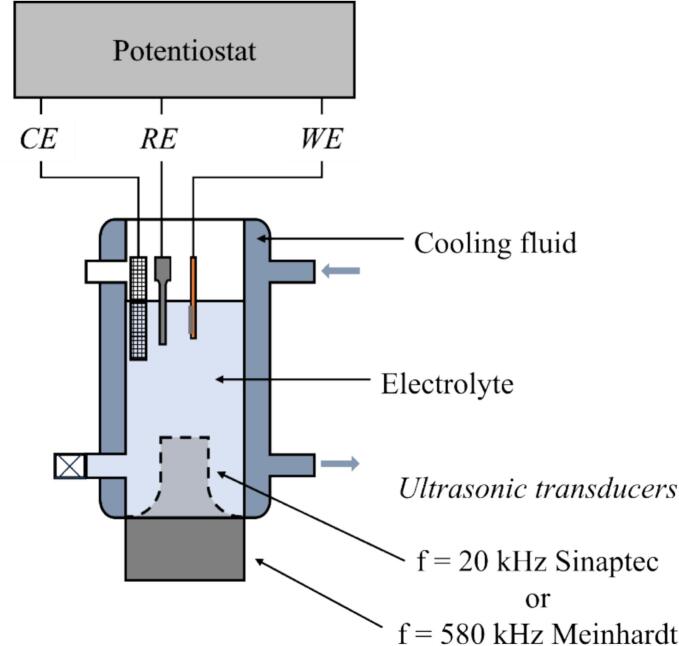
Schematic diagram of the sonoelectrochemical setup.

The sonoelectrochemical reactor (Fig. 1), was composed of a double-walled cell 500 mL (Meinhardt Ultrasonics), a thermostat bath (Neslab RTE 740), and two ultrasonic transducers (20 kHz – Sinaptec with a 20 mm diameter tip, 580 kHz – Meinhardt Ultrasonics with a 52 mm diameter plate).

To conduct electrochemical measurement, a potentiostat (Metrohm – PGSTAT204) was used in a three-electrode configuration: a platinised titanium mesh as counter electrode (CE), a saturated calomel electrode, SCE (*E*_SCE_ = +0.241 V_/SHE_ and *E*_SCE_ = *E*_SCE/SHE_ – *E*_RHE/SHE_ = +0.241 – (–0.828) = +1.069 V_/RHE_, pH 14)) as reference electrode (RE) and a working electrode (WE) prepared following an electrodeposition procedure.

### 2.1 Electrode preparation

The working electrodes used in this work were made of either flat coatings of electrodeposited nickel (Ni planar) or porous layer of electrodeposited nickel by the dynamic hydrogen bubble template method (Ni DHBT) [20]. For electrodepositing a planar nickel coating, a nickel watts bath was used and composed of 1.33 M NiSO_4_·6H_2_O, 0.21 M NiCl_2_·6H_2_O and 0.65 M H_3_BO_3_ at 60 °C. Using a copper plate as a substrate and selecting a 1x1 cm^2^ area by masking, a current density of 30 mA cm^−2^ was applied for 12 min. According to *Faraday’s Law*, these parameters allow us to obtain a thickness of 5 μm.

(5) $$n_{\mathrm{Ni}}=\frac{I.\Delta t}{z.F}$$

(6) $$e_{\mathrm{Ni}}=\frac{I.M_{\mathrm{Ni}}.\Delta t}{A.z.F.\rho_{\mathrm{Ni}}}$$

where *I* is the current (A), Δt is the electrodeposition duration (720 s), z is the number of electrons exchanged (=2), *F* is the Faraday’s constant (96,485 C.mol^−1^), A is the surface area (cm^2^), *n*_Ni_ is the quantity of electrodeposited nickel on the surface (mole), *M*_Ni_ is the atomic mass of nickel (58.7 g.mol^−1^), $\rho_{\mathrm{Ni}}$ is the density of Ni (8.9 g cm^−3^), and e_Ni_ is the thickness of planar nickel layer.

Prior to the application of the DHBT technique on the porous nickel layer, a planar nickel coating was systematically prepared. Then, to proceed on the DHBT deposition, a current density of 1 A cm^−2^ for 2 min was applied in a solution of 0.2 M NiCl_2_·6H_2_O and 2.0 M NH_4_Cl at ambient temperature. After each electrodeposition, the electrode was immerged for 10 min in deionised and deaerated water with bubbling N_2_.

### 2.2 Sonoelectrochemical reactor characterisation

Acoustic power is the transmitted energy from the transducer to the liquid volume using a calorimetric method [56], and is used as the international standard parameter to compare different sonoreactors [45,51,[57], [58], [59], [60]]. This measurement consists of ultrasonicating deionised water for 3 to 5 min at various amplitudes (from 20 % to 90 % for 20 kHz and from 40 % to 90 % for 580 kHz) in order to select the expected transmitted power.

To evaluate the mass transfer enhancement induced by ultrasound on a given location, electrochemical studies were conducted on a fast, quasi-reversible systems using the Fe(CN)_6_^−3^/Fe(CN)_6_^−4^ redox couple at low concentrations to highlight the mass transfer limitations. The electrolyte was composed of 0.01 M K_3_Fe(CN)_6_ and 0.1 M NaOH at 25 °C. Potentiostatic linear sweep voltammetry (LSV) experiments were conducted from + 1.21 V_/RHE_ to + 0.76 V_/RHE_ at a scan rate of 1 mV.s^−1^. In addition, current density was monitored vs. time (chronoamperometry − CA) to check the stability of the limiting current density (*j*_lim_) plateau. This limiting current density was subsequently used to calculate the mass transfer coefficient (*k_d_*) using Eq. (7), which has been validated in the context of sonoelectrochemistry [61,62]:

(7) $$j_{lim}=z.F.C_{Fe\left(III\right)}^{*}.k_{d}$$

where $j_{lim}$ is the limiting current density (A cm^−2^), z is the number of electrons exchanged (=1), *F* is the Faraday’s constant (96,485 C.mol^−1^), $C_{Fe\left(III\right)}^{*}$ is the concentration of Fe(III) species in the bulk electrolyte (mol cm^−3^), and *k_d_* is the mass transfer coefficient (cm.s^−1^).

### 2.3 Hydrogen performance evaluation

To evaluate the influence of ultrasound on the HER in alkaline environment, a solution of 1.0 M KOH was used as the electrolyte. At 25 °C, a linear sweep voltammogram (LSV) from + 0.1 V_/RHE_ to −0.5 V_/RHE_ at a scan rate of 0.3 mV.s^−1^ to ensure a quasi-steady state [46] was recorded using the same three-electrode configuration as for the mass transfer characterisation, followed by a galvanostatic electrochemical impedance spectroscopy (EIS) at −10 mA cm^−2^ from 10^5^ Hz to 0.1 Hz with a current density perturbation (RMS) of ± 1 mA cm^−2^. The uncompensated resistance (*R*_U_) was extracted from the EIS measurement at very high frequencies to correct the LSVs curves. All currents were normalized based on the geometric surface area (A_geo_ = 1 cm^2^).

The *IR*-corrected LSV curves were used to measure the potential at which −10 mA cm^−2^ was reached and plotted as log(*j*) vs. *η*, where *j* is the current density (A cm^−2^) and *η* is the HER overpotential (V). The latter was calculated from Eq. (8):

(8) $$\eta =E_{\mathrm{applied}}-E_{H_{2}/H_{2}O}^{\mathrm{rev}}$$

where $E_{\mathrm{applied}}$ is the applied potential (V_/RHE_) and $E_{H_{2}O/H_{2}}^{\mathrm{rev}}$ is the equilibrium or the *Nernst* potential for the HER.

The Tafel slopes (*b*) were obtained from the *IR*-corrected LSV curves with the Butler-Volmer equation (BVE) where the charge transfer (i.e. reaction kinetic) is the limiting phenomenon. As the overpotential value (η) increases, the formula can be linearized to (9):

(9) η=a+b.log(j)

where *a* is the origin value (V), and *b* is the Tafel slope (V.dec^−1^). The lower the latter parameter is, the better the performance, as it indicates that increasing the operating current density of one decade necessarily increases the potential value by *b*. Moreover, this value is crucial as it indicates the rds (rate-determining step) of the HER mechanism occurring at the electrode surface.

Finally, an equivalent circuit model (ECM) was used to characterise the different parameters in the Nyquist plot, namely the charge transfer resistance and the effective double layer capacitance [4,63].

## 3 Results and discussion

### 3.1 Roughness and porosity characterizations

**Fig. 2:**
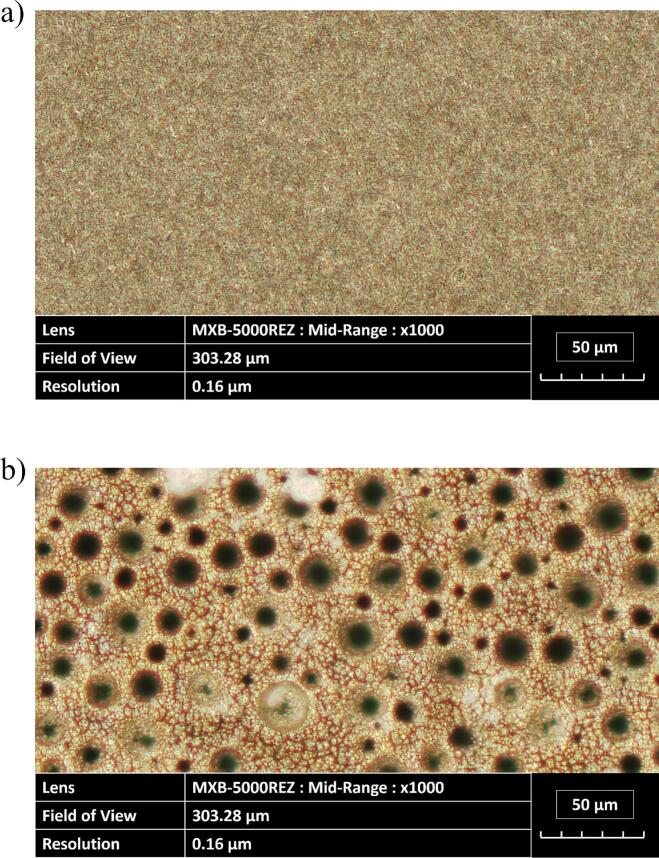
Image obtained using an optical microscope of an electrodeposited (a) planar nickel layer and (b) nickel-DHBT porous layer (Zoom: x1,000).

**Fig. 3:**
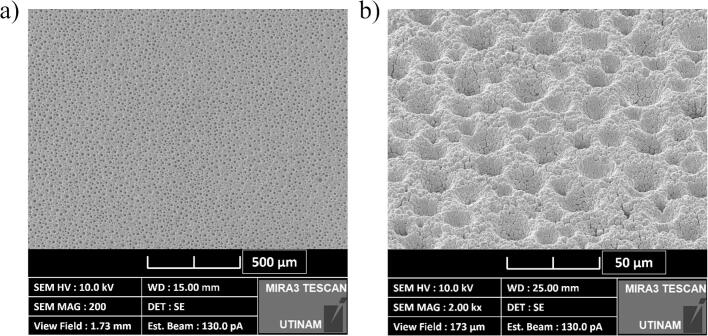
SEM images of the nickel-DHBT electrode (a) Zoom: x200, (b) Zoom: x2,000.

To observe the structure of the electrodeposited layer, optical microscopy (Fig. 2) and SEM imagery (Fig. 3) were conducted. Under classical conditions, the coatings adhered to the smooth surface pattern of the substrate (Fig. 2(a)). In contrast, electrodes made by the DHBT method led to the formation of a porous layer, which in general increases the effective surface area of the electrode. Nickel preferentially grows around the molecular hydrogen bubbles, leading to the formation of pores and ligaments of various sizes (Fig. 2(b)) [64]. Moreover, SEM images show cauliflower-like structure that forms nanopores which increases the developed and active surface areas of the electrode.

Therefore, quantitative parameters can be extracted from surface measurements [65]. The arithmetical mean height (*S*_a_) and the root mean square height (*S*_q_) parameters are commonly used metrics to assess surface roughness. However, additional parameters are required to fully characterize the surface area. Kurtosis (*S_ku_*) describes the sharpness of the probability density function of the surface profile, where higher values indicate a surface with sharper features. Skewness (*S_sk_*) measures the asymmetry of the profile relative to S_a_, where positive values indicate a surface dominated by high peaks, while negative values suggest the presence of deep valleys.

Finally, to evaluate the fractal characteristics of a surface, the power spectral density (PSD) is used to identify the spectral exponent (*β*) of the porous structure, which is associated with the fractal characteristics of the surface [66]. This value ranges between 1 and 3 for structures exhibiting fractional Brownian motion. When *β* approaches 1, the surface is increasingly dominated by fractal characteristics. Conversely, as *β* nears 3, large-scale variations become more prominent.

From planar to DHBT electrodes, the increase in *S*_a_ and *S*_q_ reflects the emergence of peaks and valleys in the surface profile, indicating a more developed surface. The evolution of *S_ku_* and *S_sk_* suggests that these peaks are relatively blunt, while the valleys are sharper. Additionally, the decrease in *β* toward a value close to 2 for the porous layer implies a greater influence of fractal structures compared to the planar surface.

### 3.2 Characterisation of the sonoelectrochemical reactor

#### 3.2.1 Volume characterization – Acoustic power

A calorimetric study was carried out for both high and low frequency ultrasonic systems at different acoustic amplitudes.

**Fig. 4:**
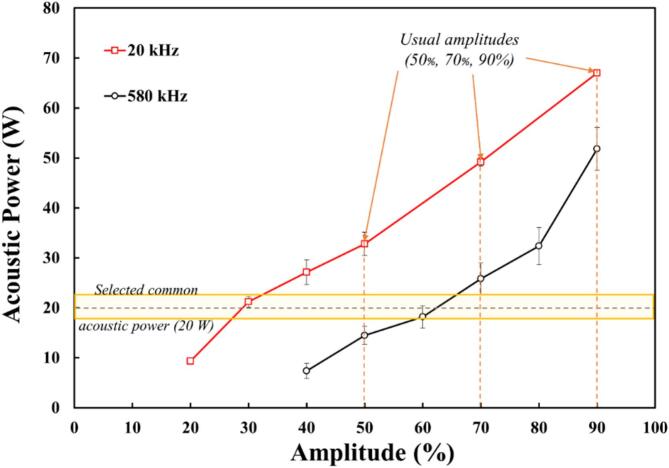
Calorimetric measurement at different ultrasonic amplitudes using the Sinaptec (20 kHz) and Meinhardt (580 kHz) ultrasonic systems.

Fig. 4 shows their respective increase as a function of the acoustic amplitudes, allowing the selection of the different acoustic powers used for further experiments.

According to Fig. 4, 20 kHz releases more acoustic energy in the volume than 580 kHz for a given acoustic amplitude, which can be explain by greater cavitation implosion at low frequencies than at high frequencies [67]. Furthermore, a range of acoustic powers is common for both setups (around 20 W – highlighted in yellow in Fig. 4) making possible a comparison at different ultrasonic frequencies for the same transmitted acoustic power. Four transmitted acoustic powers were chosen and are presented in Table 3, together with their associated acoustic amplitudes (50 %, 70 % and 90 %) to their respective transmitted acoustic powers, and one acoustic power (20 W) shared by both ultrasonic frequencies if acoustic amplitudes are adjusted.

**Table 3:** Chosen acoustic powers related to the controlled acoustic amplitudes for this study.

20 kHz		580 kHz	
Amplitude [%]	Acoustic power [W]	Amplitude [%]	Acoustic power [W]
30	20.0	50	14.5
50	32.8	63	20.0
70	49.2	70	25.8
90	67.0	90	51.8

#### 3.2.2 Surface characterization – Mass transfer

Electrochemical reactions take place at an electrode surface, meaning that most processes are surface dependent. Hydrodynamic effects influence the passage of reactants and removal of products at the electrode surface. Subsequently, the influence of ultrasound will be characterized, as it generates local turbulences, including ultrasonic wind and the collapse of asymmetric bubbles. [52,[68], [69], [70], [71]]. This characterisation serves as an indicator for the interactions between flat or porous electrodes and ultrasound, irrespective of the ultrasonic frequency and electrode-to-horn distance used.

**Fig. 5:**
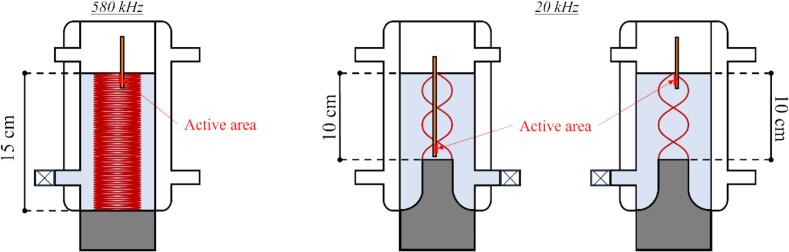
Schematic diagram of sample localizations in the various ultrasonic fields (left: 580 kHz, right: 20 kHz).

The distances between the ultrasonic transducer and the working electrode in both ultrasonic set-ups (20 kHz and 580 kHz) are represented in Fig. 5. The wavelength for 580 kHz and 20 kHz is $\lambda_{\text{20}\text{kHz}\;}=\;\text{7.5 cm}$ and $\lambda_{\text{580}\text{kHz}\;}=\;\text{2.6 mm respectively}$, yielding a totally different cavitation intensity distribution [41]. From this distribution, the electrode was positioned at the liquid surface (h = 15 cm) at 580 kHz. Given that the wavelength is significantly smaller than the electroactive area (1 x 1 cm^2^), the behaviour of the liquid at varying depths of the electrode surface is relatively uniform. In contrast, at 20 kHz, the wavelength is larger than the electrode height, implying that the depth influences the local cavitation activity on the surface. In addition, the distribution of acoustic energy is different when the 20 kHz horn, with a diameter smaller than the corresponding wavelength in water, produces a divergent field, while the 580 kHz plate transducer, larger than the wavelength at this frequency, generates a more Fresnel field before diverging close to the water/air interface [48]. Therefore, two positions were selected for this ultrasonic frequency: one at the liquid surface (h = 10 cm) which acts as a free interface (overpressure node), and another closer to the transducer (h = 3.5 cm), where the overpressure is higher, thereby enhancing cavitation activity.

**Fig. 6:**
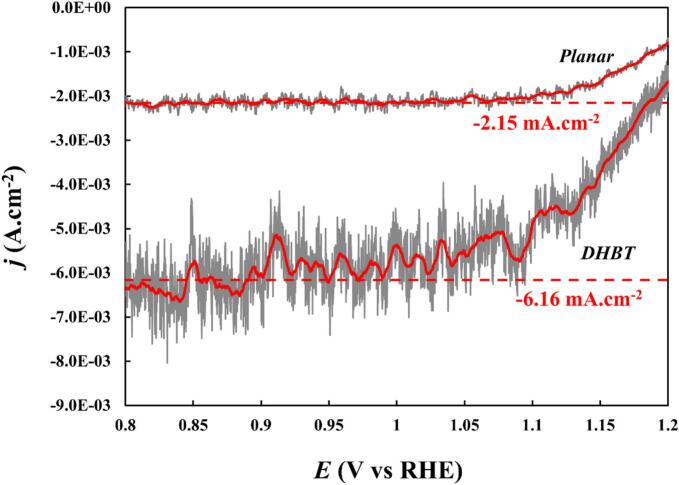
Examples of LSV curves for mass transfer characterisation in 0.01 M K_3_Fe(CN)_6_ and 0.1 M NaOH at 25 °C and a scan rate of 1 mV.s^−1^ for both electrodes at 580 kHz – 20 W.

Ultrasound induces hydrodynamic convection at the electrode surface caused by cavitation or acoustic streaming. The dominant mechanism is strongly frequency dependent. Lower frequencies like 20 kHz promote transient cavitation, while higher frequencies (i.e. 580 kHz) promote acoustic streaming [72,73]. The LSV curve for mass transfer measurements therefore much similar to the behaviour observed with a rotating disc electrode (RDE). Fig. 6 presents an example of analysis following the method described by Islam *et al.* [62]. The pronounced current spikes are attributed to cavitation implosions occurring at the electrode surface [62,[74], [75], [76]]. By smoothing the curves, the plateau region reveals the mass-transfer-limited current density, $j_{lim}$, which is subsequently used to calculate the mass transfer coefficient, $k_{d}$, according to the procedure outlined in section 2.2.

**Fig. 7:**
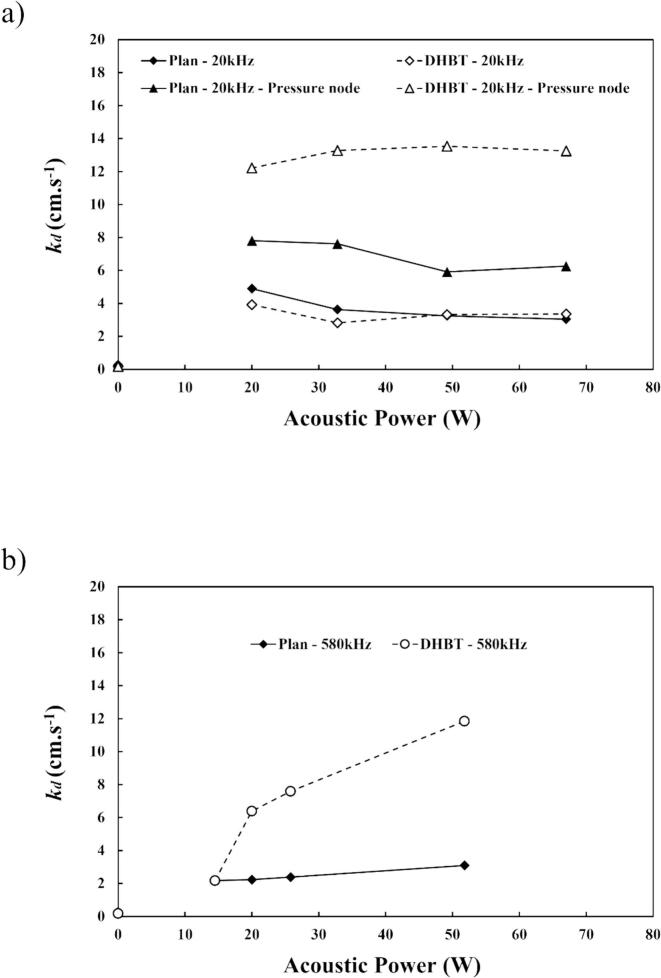
*k*_d_ measured from LSV curves for 0.01 M K_3_[Fe(CN)6] in 0.1 M of NaOH at 25 °C (a) *f* = 20 kHz – d = 10 cm and d_PN_ = 3.5 cm; (b) *f* = 580 kHz – d = 15 cm, using planar and porous (DHBT) Ni electrodes.

Fig. 7 shows the mass transfer coefficient (*k*_d_) measured for each ultrasonic operating conditions related to their respective acoustic powers for (a) 20 kHz at 3.5 cm (pressure node) and 10 cm and (b) 580 kHz at 15 cm. At 20 kHz and 10 cm, the stirring effect of ultrasound is mostly due to ultrasonic wind (convection) and is identical between the planar and DHBT electrodes. For the two other conditions, asymmetric collapse is dominant. However, the results show that mass transport reaches a limit where an increase of the acoustic power no longer has an effect.

At 20 kHz and 3.5 cm, extreme cavitating conditions exist thus maximising its local stirring. At 580 kHz, increasing the acoustic power enhances mass transport proportionally. Moreover, the combination “roughness-ultrasound” is notable in both cases where the slope is greater for the porous electrode. The difference of mass transport threshold (20 kHz) and slope values (580 kHz) between the planar and the DHBT electrode could be explained due to the increase of electroactive area is enhanced by the acoustic stirring in disrupting the diffusion layer, improving the reactant supply from the bulk to the surface.

In any case, this mapping of mass transfer values allows to link this parameter (*k_d_*) with the efficiency of electrolysis.

### 3.3 Synergy between a porous electrode and ultrasound on HER

#### 3.3.1 Potential at –10 mA cm^−2^

**Fig. 8:**
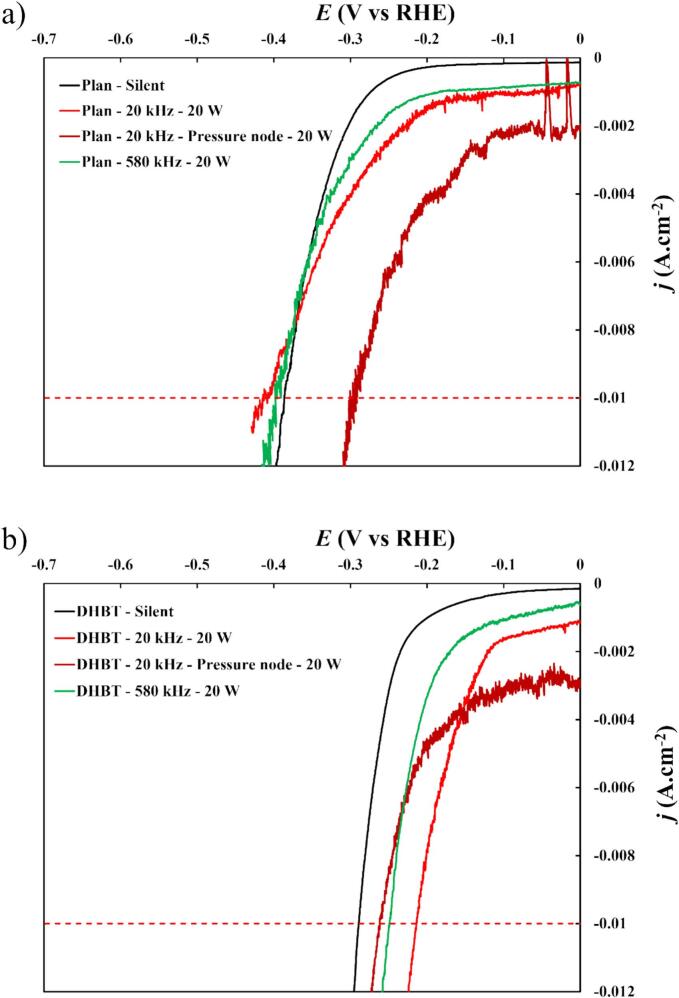
LSV *IR*-corrected curves in HER region in 1.0 M KOH at 25 °C and a scan of 0.3 mV.s^−1^, for (a) a planar nickel electrode, and (b) a DHBT nickel electrode. The acoustic power was fixed at *P*_ac_ = 20 W, at different ultrasonic frequencies (20 and 580 kHz) and distances between WE and the sonotrode (10 and 3.5 cm for 20 kHz, 15 cm for 580 kHz).

To achieve a specific current density (*j*), the effective potential serves as a reliable indicator of power losses during electrolysis, as the electrical power (*P*) input is the product of the current (*I*) and the cell voltage (*U*_cell_) during the electrolysis process. In this study, the standardised current density (*j*) chosen was –10 mA cm^−2^. Fig. 8 illustrates the potential at −10 mA cm^−2^ (${\text{E}}_{\text{-10mA.c}{\text{m}}^{\text{-2}}}$) derived from LSV measurements in the four conditions described previously.

Under *silent conditions*, the overpotential was found to be −386 mV_/RHE_ which is in fairly good agreement with the value obtained by Foroughi *et al.* [6]. Transitioning from planar to DHBT electrodes led to an increase in surface area, resulting in a reduced overpotential of −289 mV_/RHE_ at −10 mA cm^−2^ (25 % decrease), indicating enhanced HER performances. This improvement can be attributed to the porous structure of the DHBT electrodes, which increases the number of active sites. As a result, local resistances and current densities are reduced, leading to more uniform charge transfer at the interface, thus decrease in overpotential.

**Fig. 9:**
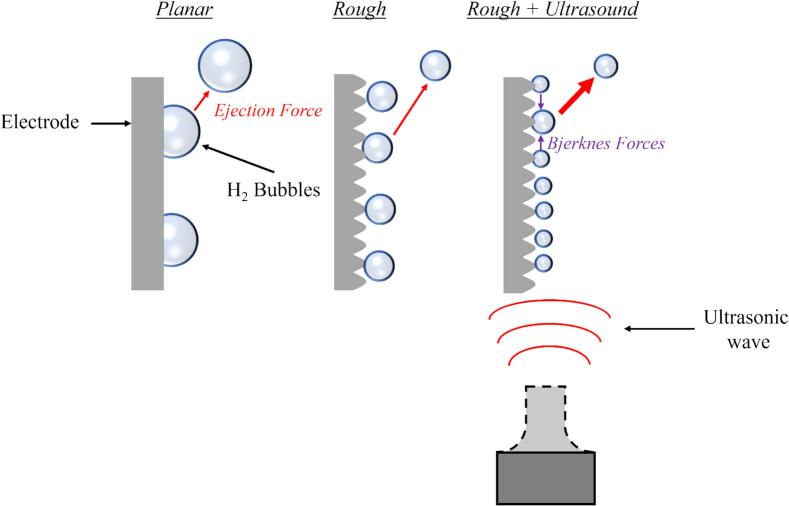
Diagram illustrating the effects of a rough surface and ultrasound on bubble size, showing the various forces acting on the bubble.

Under ultrasonic conditions, ${\text{E}}_{\text{-10mA.c}{\text{m}}^{\text{-2}}}$ move towards less negative values, although even if changes are little in comparison to the clear benefits from DHBT vs. planar electrodes (Fig. 8). This reduction in overpotential is likely to be a result of the efficient removal of gas bubbles, which lessens the inhomogeneity of the current density across the surface caused by bubble adhesion. Ultrasonication during HER is known to effectively remove bubbles through Bjerknes forces, reducing their size [77,78]. This process ultimately lowers the ohmic drop and improves the renewal of reactants at the surface (Fig. 9).

However, as it can be seen in these LSVs under ultrasonication, a non-zero constant current density is noticeable before the HER region, where normally no faradaic reactions should occur. In this region, nickel should be in metallic state where no oxidation had occurred prior to the experiment. Thus, the current density is mainly due to non-faradaic reactions such as the adsorption/desorption of reactants.

Under ultrasonication conditions, there are two phenomena occurring during acoustic cavitation: (i) the implosion of cavitation bubble which generates shockwaves that erode and disrupt the diffusion layer, and (ii) the production of radicals (H^•^ and OH^•^) which can generate hydrogen peroxide (H_2_O_2_) and H_2_, a process known as water sonolysis [79].

In order to separate both processes, we conducted LSV measurements in HER region in which H_2_O_2_ was injected in the solution at fixed time intervals to simulate H_2_O_2_ production (see *Supplementary Materials – A.1l*). It was found that H_2_O_2_ does not appear to generate a significant current density before the HER region.

As the transducer was electrically isolated from the measurement setup (no leakage current) and directly in contact with the electrolyte, the explanation for these unexpected current densities could be that the shockwaves of cavitating bubbles renders difficult the adsorption of reactants at the surface electrode. Similarly to the mass transfer curves in Fig. 6, the disturbances in current densities originate from the acoustic cavitation [62,[74], [75], [76]]. It should also be noted that shockwaves are stronger at 20 kHz than at 580 kHz, which could explain the differences in values of initial current densities and fluctuations.

**Fig. 10:**
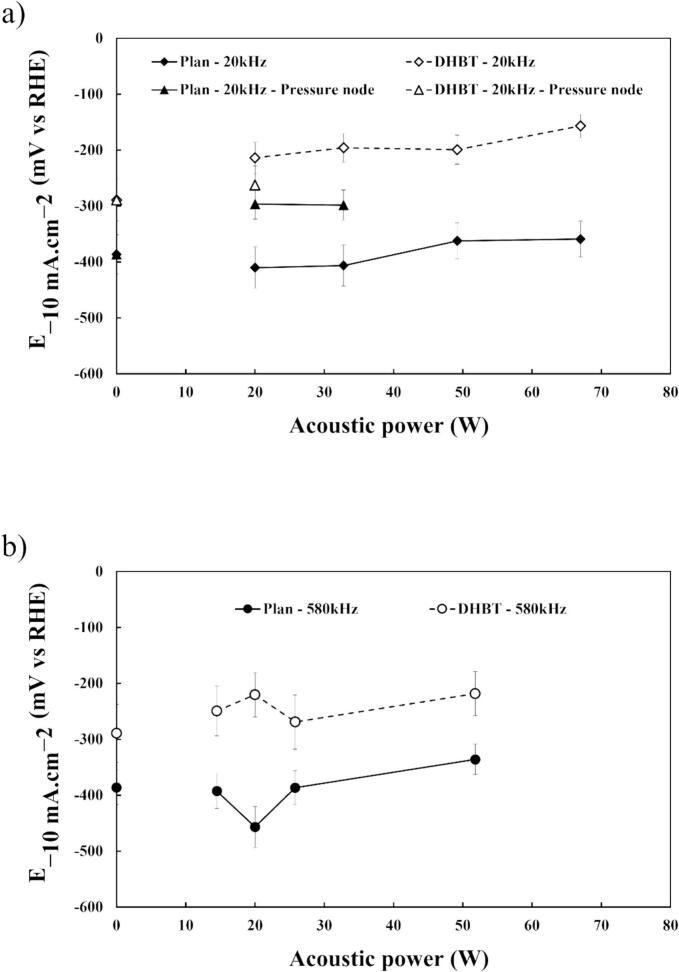
Potentials values at −10 mA cm^−2^ from *IR*-corrected LSVs vs. acoustic power (a) at 20 kHz and (b) at 580 kHz, in HER region in 1.0 M KOH at 25 °C using a planar or porous (DHBT) Ni electrodes.

Regarding the overpotential, Fig. 10(a) and (b) show that ${\text{E}}_{\text{-10mA.c}{\text{m}}^{\text{-2}}}$ decreases as the acoustic power increases, indicating that HER performances is enhanced by ultrasonication. This trend is consistent for both planar and porous electrodes.

**Fig. 11:**
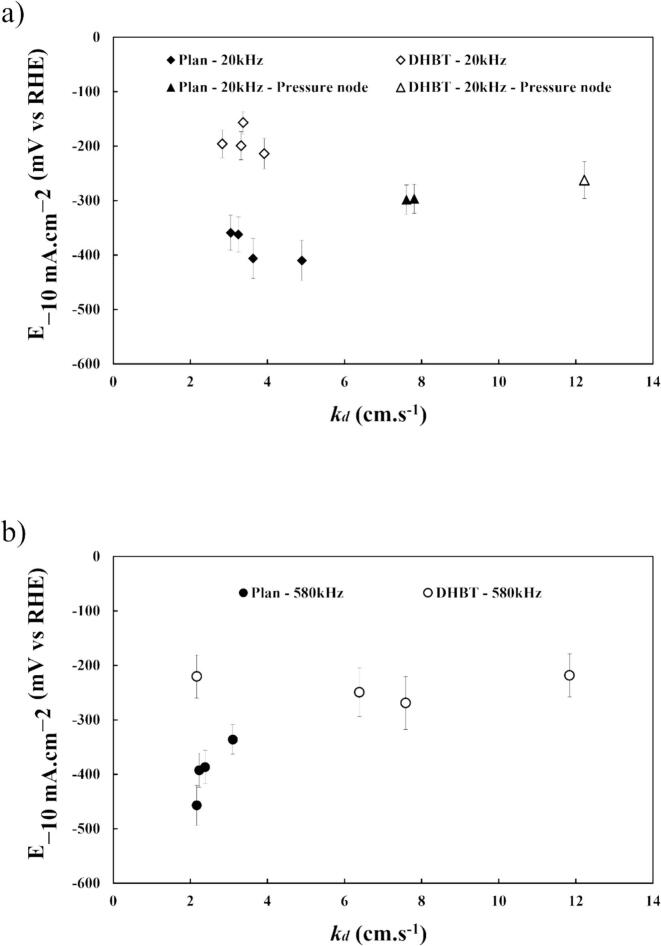
Potentials values at −10 mA cm^−2^ from *IR*-corrected LSVs vs. mass transfer coefficient *k*_d_ (a) at 20 kHz and (b) at 580 kHz in HER region in 1.0 M KOH at 25 °C using a planar or porous (DHBT) Ni electrodes.

To quantify the dependence of ${\text{E}}_{\text{-10mA.c}{\text{m}}^{\text{-2}}}$ to local stirring, potentials are plotted vs. *k_d_* (Fig. 11(a) and (b)) and different behaviours can be observed.

First, for a planar electrode at 20 kHz, increasing the mass transfer coefficient, i.e. by increasing the transmitted acoustic power or by moving the electrode from an overpressure node to an antinode, appears to shift only slightly the potential to less negative values. This mean that the ultrasonic activity is barely linked to the cavitation intensity. For the porous electrode, this shift is greater i.e. the potential required to reach −10 mA cm^−2^ is reduced for low *k_d_* values. Indeed, the ultrasonic efficiency is better for low acoustic powers if the surface roughness is high. At higher acoustic powers, a degradation of the DHBT surface may occurs (section 3.3.4). At 580 kHz with the electrode positioned at 15 cm, increasing the agitation modify the efficiency for the flat surface, but does not appear to modify significantly the overpotential in the case of DHBT coatings. Eventually, at both ultrasonic frequencies and in the case of surfaces with high roughness levels, bubble removal is more effective as far as ultrasound is active. Even at low *k_d_*, ultrasonic waves at the interface promotes the ejection of bubbles without requiring excessive energy.

#### 3.3.2 Tafel slope

**Fig. 12:**
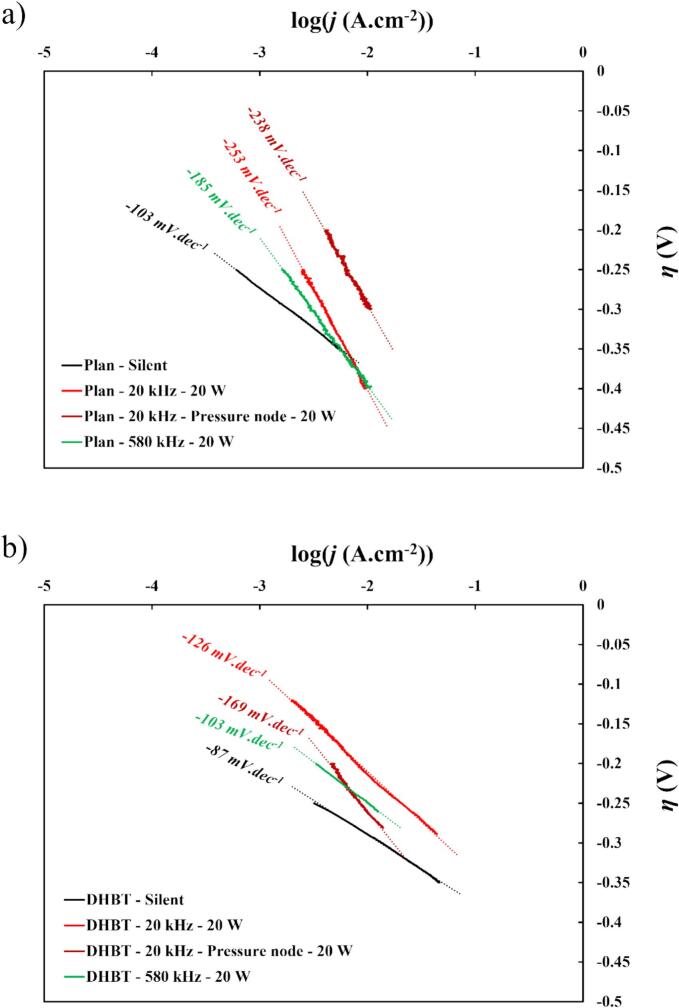
Tafel slopes derived from the previous LSV curves in HER region in 1.0 M KOH at 25 °C, for (a) flat nickel electrodes, and (b) DHBT nickel electrodes. The acoustic power was fixed at *P*_ac_ = 20 W, at different frequencies (20 and 580 kHz) and distances between WE and the sonotrode (10 and 3.5 cm for 20 kHz, 15 cm for 580 kHz).

In this study, lower Tafel slope values were observed for both planar and porous electrodes Fig. 12. The values obtained for Plan and DHBT, −103 mV.dec^−1^ and −87 mV.dec^−1^ respectively, both fall within the range where the rate-determining step (rds) of the mechanism is between the Volmer and the Heyrovsky steps (between −120 and −40 mV.dec^−1^). In this case, the increase in active surface simply alleviates the limitation of the interaction between adsorbed hydrogen atoms (H_ads_) and OH^–^ across the entire surface area, thus explaining the shift of Tafel slope value toward the Heyrovsky step.

However, ultrasonication appears to have a negative effect on the reaction mechanisms. At 20 kHz, the Tafel slope increases with both electrodes, with the negative effect being more pronounced as the electrode is positioned closer to the sonotrode. Conversely at 580 kHz, electrode electrocatalytic properties are less affected by ultrasound, especially in combination with DHBT Ni.

**Fig. 13:**
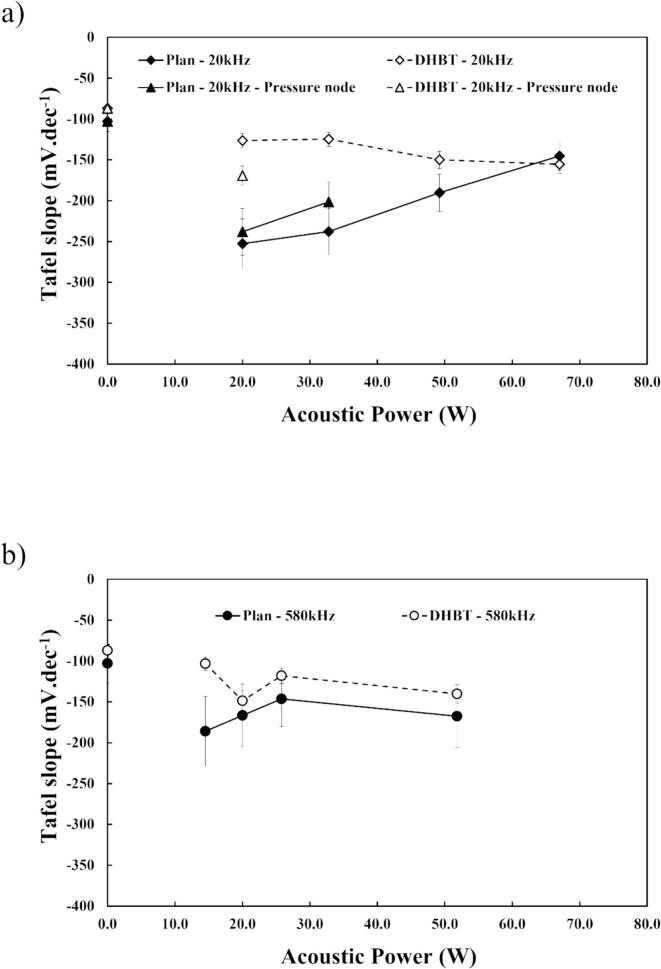
Tafel slopes (*b*) values vs. acoustic power (a) at 20 kHz and (b) at 580 kHz in HER region in 1.0 M KOH at 25 °C using planar or porous (DHBT) Ni electrodes.

As illustrated in Fig. 13(a) and (b), starting ultrasonic irradiation led to an increase of the Tafel slope values, illustrating negative impacts compared to *silent* conditions. The values with ultrasound are above the Heyrovsky step range (< −120 mV.dec^−1^), meaning that the rds has shifted to the Volmer step. This suggests that ultrasound may alter the HER mechanism. However, this contradicts previous studies, which observed no significant modification of the mechanism [6,36,44,78]. This discrepancy could imply that other reactions are occurring during HER or the adsorption of water molecules are greatly disturbed, influencing the overall mechanism.

Looking back on the experiment with the injection of H_2_O_2_, no influence of the H_2_O_2_ was found on HER mechanism which is in agreement with previous works suggesting that radicals produced by sonolysis does not modify the mechanism [6,36,78]. The Volmer step corresponds to the water adsorption mechanism, indicating that the increase due to ultrasonication could mean that the strong mechanical convection due to acoustic shockwaves could hinder the water adsorption at the electrode surface. This explanation corroborates with the apparition of the unexpected current density in non-faradaic region detailed in section 3.3.1.

**Fig. 14:**
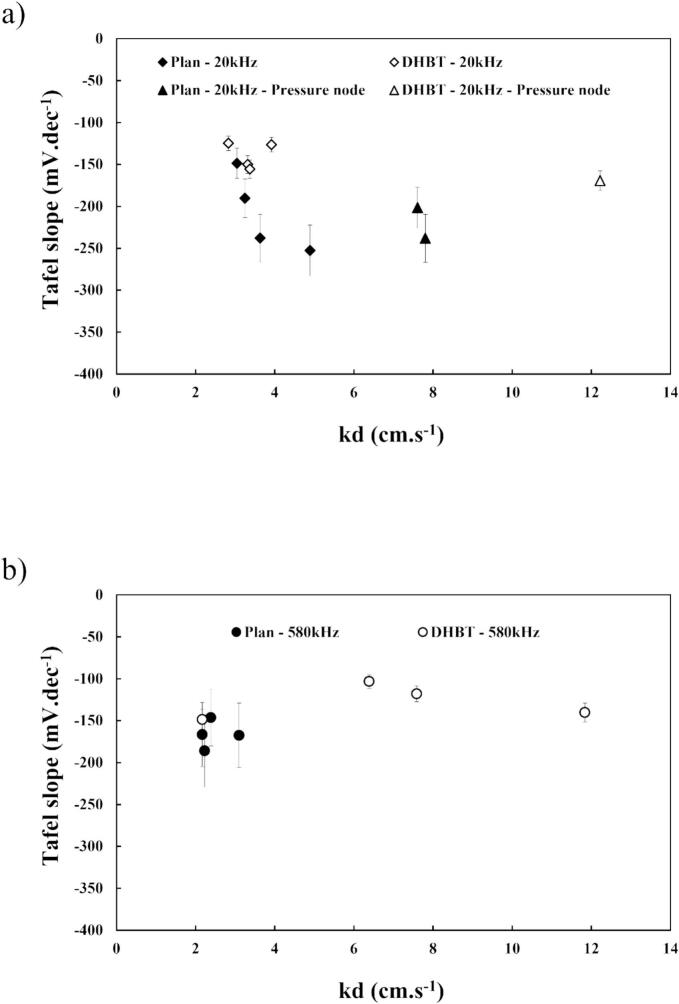
Tafel slopes values vs. mass transfer *k_d_* (a) at 20 kHz and (b) at 580 kHz in HER region in 1.0 M KOH at 25 °C, using flat or porous (DHBT) Ni electrodes.

Regarding the influence of mass transfer (Fig. 14(a) and (b)), it is difficult to establish a global pattern, but several conclusions can be drawn.

Once again, it is possible to make a relationship between low mass transfer i.e. little and controlled ultrasonic agitation, due to the obtained smallest Tafel slopes at low *k*_d_, especially with high roughness (DHBT Ni). If the 580 kHz experiments do not see too much Tafel slope values changes vs. mass transfer increase, the 20 kHz situation is different with an obvious degradation as soon as the agitation is above the 4 cm.s^−1^ threshold.

Thus, the increases in Tafel slopes can be attributed to the effects of shockwaves from acoustic cavitation at the electrode surface, which may alter the electrochemical reaction mechanisms by hindered the adsorption of water molecules. However, further investigation is necessary to fully elucidate the changes in the reaction mechanisms in order to improve the coupling of “ultrasonic frequency/electrode roughness”.

#### 3.3.3 AC impedance at −10 mA cm^−2^

**Fig. 15:**
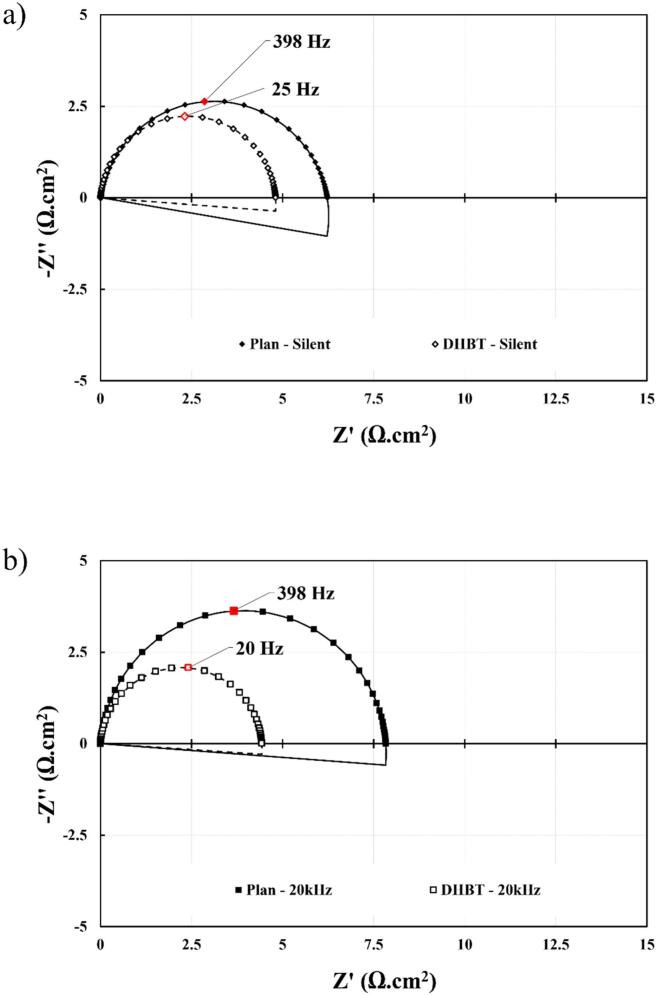

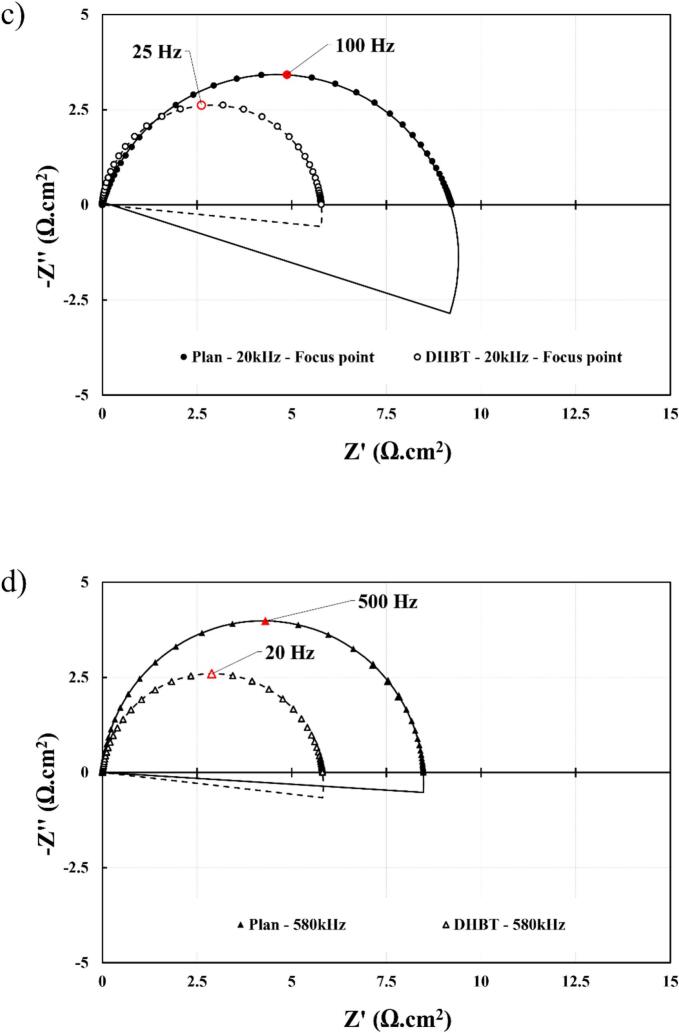
Nyquist plots of the ECM (*R*_u_ subtracted) of HER in 1.0 M KOH using Ni electrodes at 25 °C and 20 W of acoustic power: (a) *silent conditions*, (b) 20 kHz – 10 cm, (c) 20 kHz – 3.5 cm (Pressure node), and (d) 580 kHz – 15 cm. Measurement at −10 mA cm^−2^ from 10^5^ Hz to 0.01 Hz and an RMS amplitude of ± 1 mA cm^−2.^

EIS measurements were performed to evaluate the synergetic effects of DHBT with ultrasound. In this study, *R*_ct,eff_ (Ω.cm^2^) and *C*_dl_ (F) are the two parameters of interest, where the former is linked to the reaction kinetic, and the latter to the electrochemically active surface area (ECSA). As shown in Fig. 15, *R*_ct,eff_ is determined by the diameter of the semi-circle, and *C*_dl_ is obtained from the values of the CPE and *R*_ct,eff_ for which each variable is deduced from the electrical circuit model (ECM). The chosen ECM of this study is a *R*_u_ – *R*_ct_/CPE circuit, where *R*_u_ is the resistance caused by the ohmic drop from the distance between the WE and RE.

By analysing the R/CPE part of the ECM, the effective *C*_dl,eff_ and *R*_ct,eff_ (Table 4) have been extracted at various ultrasonic operating conditions. Under *silent* conditions, the DHBT *C*_dl,eff_ is significantly higher than that of its planar counterpart. This increase is attributed to the presence of nano and micropores (Fig. 3), which expand the actual surface area available for electrochemical activity. Furthermore, ultrasonication did not affect this parameter, except at the pressure node where degradation occurred (section 3.3.4).

**Table 4:** Double layer capacitances and charge transfer resistances at 20 kHz and 580 kHz in HER region in 1.0 M KOH at 25 °C, using a flat (planar) and porous (DHBT) Ni electrodes.

**Frequency (kHz)**	**0**			**20**															**580**											
**Distance (cm)**	−			*10 cm*												*3.5 cm*			*15 cm*											
***P*_ac_ (W)**	−			20			32.8			49.2			67			20			14.5			20			25.8			51.8		

	***Planar Nickel Electrode***																													
***k*_d_ (cm.s^−1^)**	−			4.89			3.63			3.24			3.05			7.8			2.23			2.17			2.38			3.1		
***C*_dl_ (μF)**	71.5	±	17.9	55.0	±	27.0	129.0	±	63.2	102.0	±	50.0	62.2	±	30.5	158.0	±	77.4	57.9	±	17.9	36.8	±	11.4	67.5	±	20.9	68.4	±	21.2
***R*_ct_ (Ω)**	6.23	±	0.8	7.8	±	0.8	8.73	±	0.9	8.2	±	0.8	6.6	±	0.7	9.2	±	0.9	6.7	±	0.4	8.5	±	0.5	6	±	0.4	6.1	±	0.4

	***DHBT Nickel Electrode***																													
***k*_d_ (cm.s^−1^)**	−			3.92			2.83			3.32			3.37			12.22			6.38			2.17			7.59			11.84		
***C*_dl_ (mF)**	1.38	±	0.06	1.63	±	0.42	1.70	±	0.44	1.54	±	0.40	1.61	±	0.42	1.22	±	0.32	1.27	±	0.33	1.38	±	0.36	1.20	±	0.31	1.57	±	0.41
***R*_ct_ (Ω)**	4.81	±	0.34	4.43	±	0.22	4.66	±	0.23	4.64	±	0.23	4.74	±	0.24	5.78	±	0.29	4.32	±	0.13	5.82	±	0.17	4.31	±	0.13	5.32	±	0.16

As previously discussed in this section, *R*_ct,eff_ is associated with the reaction kinetics with the Eq. (10), where R is the ideal gas constant (8.314 J.K^−1^.mol^−1^), T is the temperature (K), n is the number of exchanged electrons during the reaction (z = 2), *F* is Faraday’s constant (96,485 C.mol^−1^), *j*_0_ is the exchanged current density (A cm^−2^), k is the rate constant, C is the reactant concentration (mol.m^−3^) and m is the reaction order:

(10) $${\text{R}}_{\text{ct,eff}}=\frac{\text{R.T}}{\text{z.F.}{\text{j}}_{\text{0}}}$$

(11) $${\text{j}}_{\text{0}}\;\text{= z.F.k.}{\text{C}}^{\text{m}}$$

According to the results in Table 4, *R*_ct,eff_ seems unaffected by ultrasonication, with the exception of the focal point at 20 kHz, where surface degradation is observed (section 3.3.4). By comparing this resistance between the two electrode types, the kinetic activity of the DHBT electrode is approximately 1.5 times greater, a finding which is in good agreement with the enhanced electrocatalytic properties of nickel, as discussed in 3.3.1, 3.3.2.

Therefore, the EIS measurements demonstrate that DHBT electrodes exhibits superior electrochemical properties compared to planar electrodes. Furthermore, ultrasonication does not significantly impact either the ECSA or the reaction kinetics, provided that the electrode is not positioned near the sonotrode, where strong acoustic erosion takes place.

#### 3.3.4 Degradation

In this part, the focus was on short-term degradation of the samples due to ultrasonication. When a cavitation collapse occurs near a surface, the inhomogeneous environment causes the collapsing bubble to be directed towards the surface in a process known as “microjet”. The impact of this collapse has been measured to reach pressures of up to 1,000 MPa, which velocities ranging from 70 to 180 m.s^−1^ [80]. These conditions can create pinholes in materials like polymers and even metals, a phenomenon known as acoustic erosion [81,82]. The damage causes by the erosion depends upon the type of surface material, the ultrasonic frequency and acoustic power [83]. In the case of planar nickel electrodes, the material is hard enough to withstand acoustic cavitation [6,84]. However, the porous structure of the DHBT electrode can reduce its resistance to erosion, thus it is important to survey the surface integrity after each experiment.

**Fig. 16:**
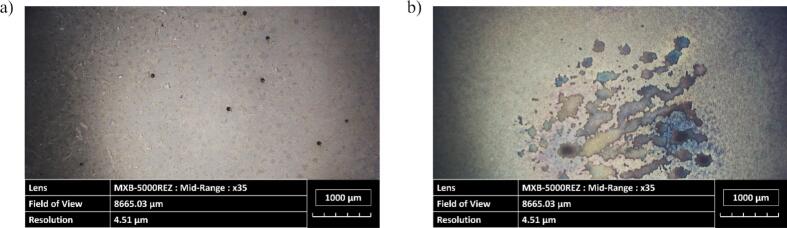
Optical microscopy images (Zoom: x35) of the effects of erosion on (a) planar and (b) DHBT electrodes after ultrasonication (30 min) at 20 kHz, pressure node and *P*_ac_ = 20 W.

Fig. 16 illustrates the surface conditions before and after 30 min of ultrasonication for both coating types. For the Ni planar coating (Fig. 16(a)), darker spots are clearly visible on the surface, indicating the influence of acoustic cavitation. However, the surface integrity appears to be largely unaffected. In contrast, for the Ni DHBT coating, the porous structure is more susceptible to ultrasonication, with the porous layer of the coating having broken down (Fig. 16(b)).

**Fig. 17:**
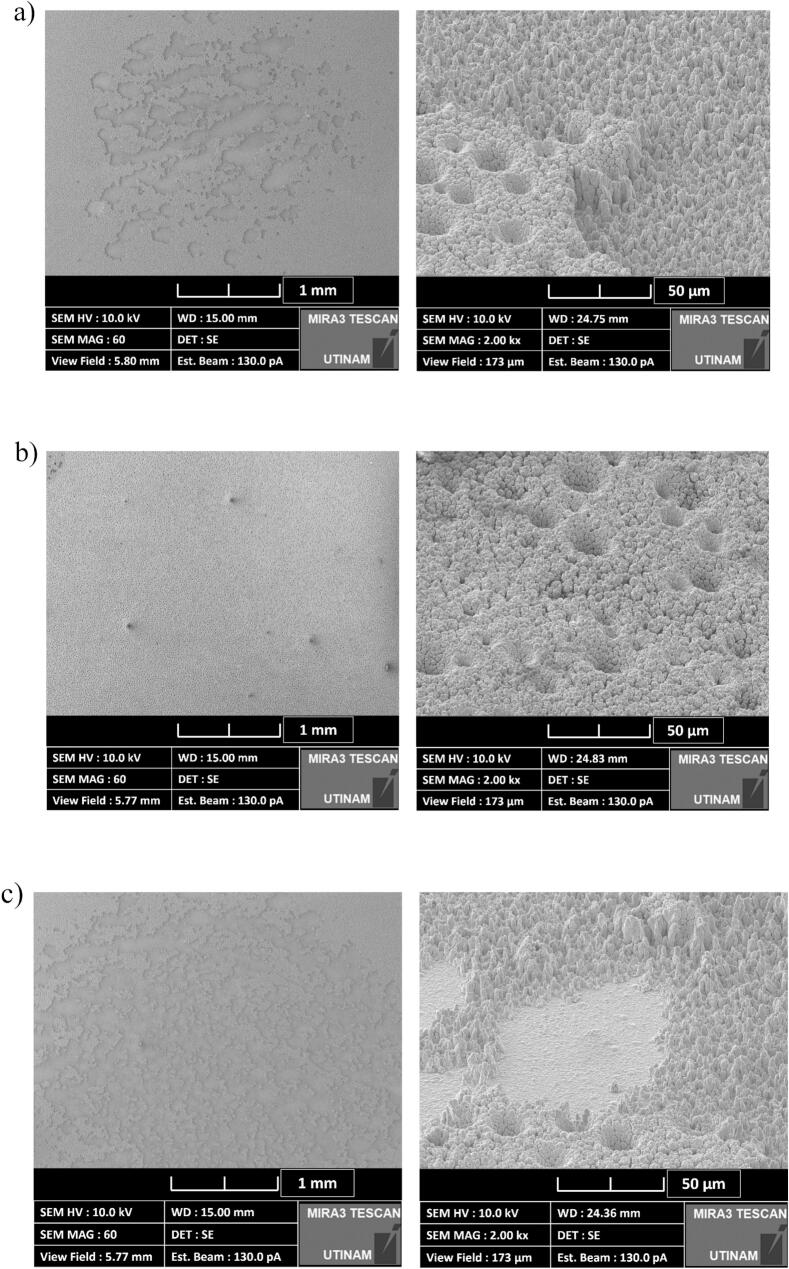

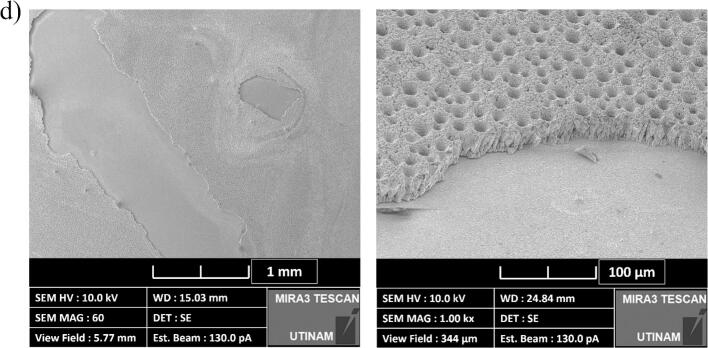
SEM images of the DHBT electrode erosion at various ultrasonic conditions after 30 min of exposure (a) 580 kHz – 14.5 W, (b) 20 kHz – 67.0 W, (c) 20 kHz – 32.8 W, and (d) 20 kHz PN – 20 W (Zoom: Left x60; Right x2,000 at 55°).

To further investigate the degradation behaviour, SEM imaging was performed, as shown in Fig. 17. At 580 kHz and 14.5 W (Fig. 17(a)), the porous structure was broken, but the layer remained intact, with spikes still visible on the surface. In contrast, at 20 kHz, the porous coating layer was completely removed leaving the planar nickel layer exposed and unaffected (Fig. 17(b) to (d)). Since this study focused on the short-term degradation of the electrode, it should be noted that industrial applications may face long-term degradation of the entire system due to prolonged ultrasonication. Shchukin *et al*. [83] have reviewed the effects of acoustic cavitation on solid surfaces [83]. Additionally, planar nickel is known to be resistant to short-term erosion; however, fatigue cracks are likely to develop over time during ultrasonication [84]. Long-term ultrasonic treatment under real conditions will be explored in future studies.

## 4 Conclusions

In this study, a sonoelectrochemical reactor operating at two ultrasonic frequencies (20 kHz and 580 kHz) was characterized through calorimetric and mass transfer coefficient *k_d_*, measurements in order to link ultrasonic effects in the bulk solution and at the electrode surfacer, whatever its localization into the reactor. Then, for the first time, the performance of an electrodeposited porous nickel coating (DHBT nickel electrode) was examined under ultrasonic conditions. HER was then performed under ultrasonication at various acoustic frequencies, powers and *k_d_*.

DHBT Ni electrodes exhibited higher overall electrocatalytic performances compared to its planar counterpart. The combination of ultrasound and surface roughness shows synergistic effects, evident from the ${\text{E}}_{\text{-10mA.c}{\text{m}}^{\text{-2}}}$ values, where bubble ejection efficiency effectively reduces it. Regarding the Tafel slope, the intense convection resulting from acoustic cavitation at the electrode surface appears to impede the water adsorption mechanism (Volmer step), which contrasts with findings from previous studies. On a positive note, the results suggest that higher ultrasonic energy input is necessary to achieve improved performance. In this study, the combination of ultrasound and surface roughness was not fully optimized, but the findings are promising. Notably, the DHBT–580 kHz pairing emerged as the most effective in our experiments. Further research is needed to determine the optimal combination of ultrasonication and roughness-controlled electrodes. Additionally, the long-term impact of ultrasonication remains an open question that requires thorough examination.

## Declaration of generative AI and AI-assisted technologies in the writing process

During the preparation of this work, the author(s) used ChatGPT in order to refine and improve the clarity of the manuscript's writing. After using this tool/service, the author(s) reviewed and edited the content as needed and take(s) full responsibility for the content of the publication.

## CRediT authorship contribution statement

**Jérémy Gravelle:** Validation, Writing – review & editing, Methodology, Writing – original draft, Investigation. **Vanessa Avramovic:** Investigation, Methodology. **Loïc Hallez:** Software, Investigation. **Jean-Yves Hihn:** Supervision, Writing – review & editing, Resources, Validation, Methodology. **Bruno G. Pollet:** Validation, Methodology, Supervision, Writing – review & editing, Resources.

## Declaration of competing interest

The authors declare that they have no known competing financial interests or personal relationships that could have appeared to influence the work reported in this paper.
