# Supplementary material for: Effects of a perpendicular ultrasonic field on planar and porous electrodes for hydrogen production in alkaline conditions
Source: Ultrason Sonochem. 2025 Jul 27;120:107481. doi: 10.1016/j.ultsonch.2025.107481 (PMC12329300; doi:10.1016/j.ultsonch.2025.107481)
Supplement: Supplementary Data 1 [file mmc1.docx]

**A.I. Measurements of the HER performance with H_2_O_2_ injection each 5 min**


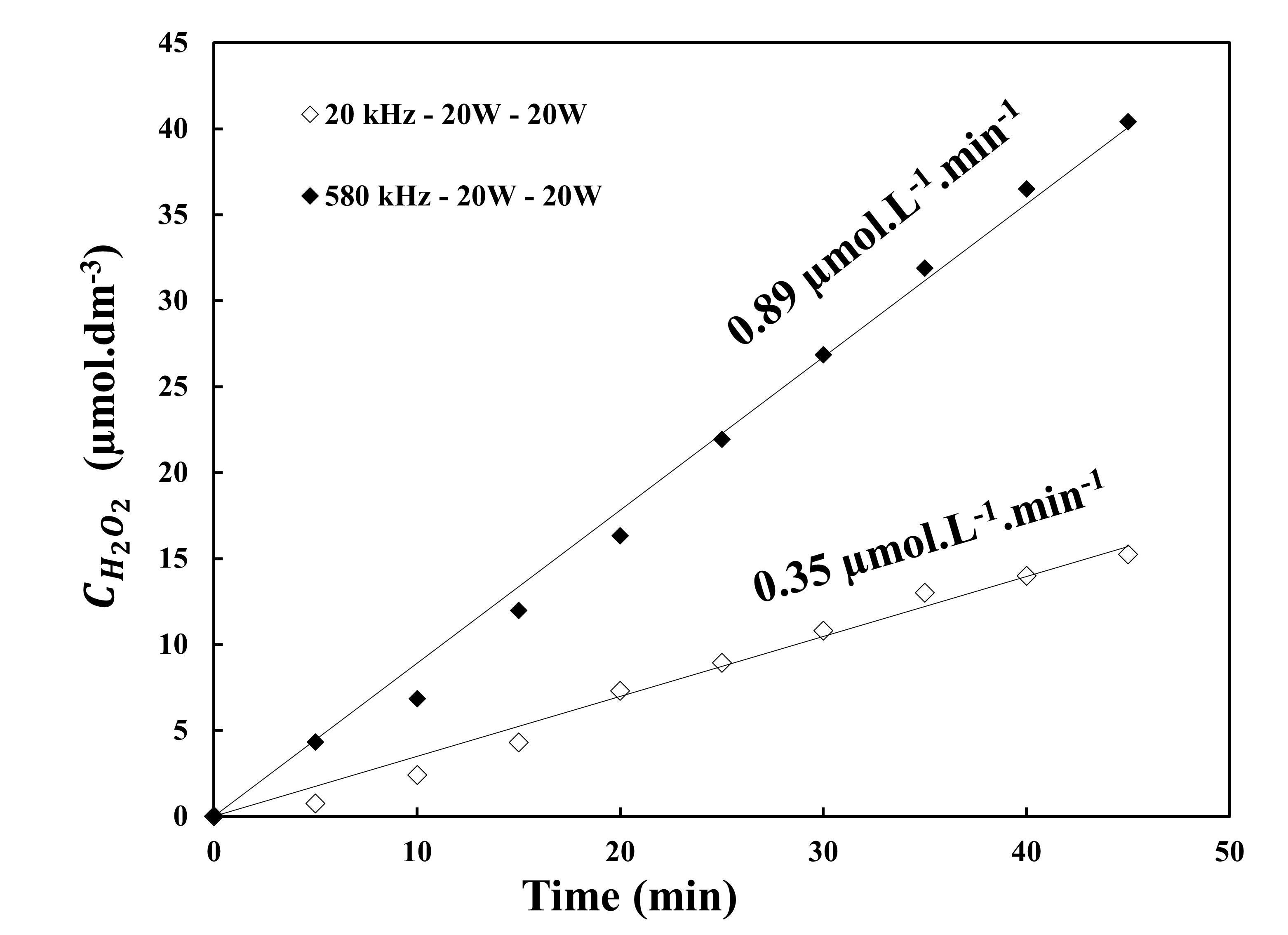


Figure A1 : Dosimetry measurements (Weissler) at 20W for both frequencies

In this experiment, dosimetry measurements were conducted at 20 W for both frequencies (Figure A1). The corresponding production rates were 0.35 µmol.L^-1^.min^-1^ at 20 kHz and 0.89 µmol.L^-1^.min^-1^ at 580 kHz. To isolate the influence of H_2_O_2_ on the HER mechanism, a 2 mmol.L^-1^ H_2_O_2_ solution was prepared. A LSV was performed in the HER region using a planar nickel electrode. During this measurement, 500 µL of the H_2_O_2_ solution was injection every 5 minutes (Figure A2 and Figure A3).


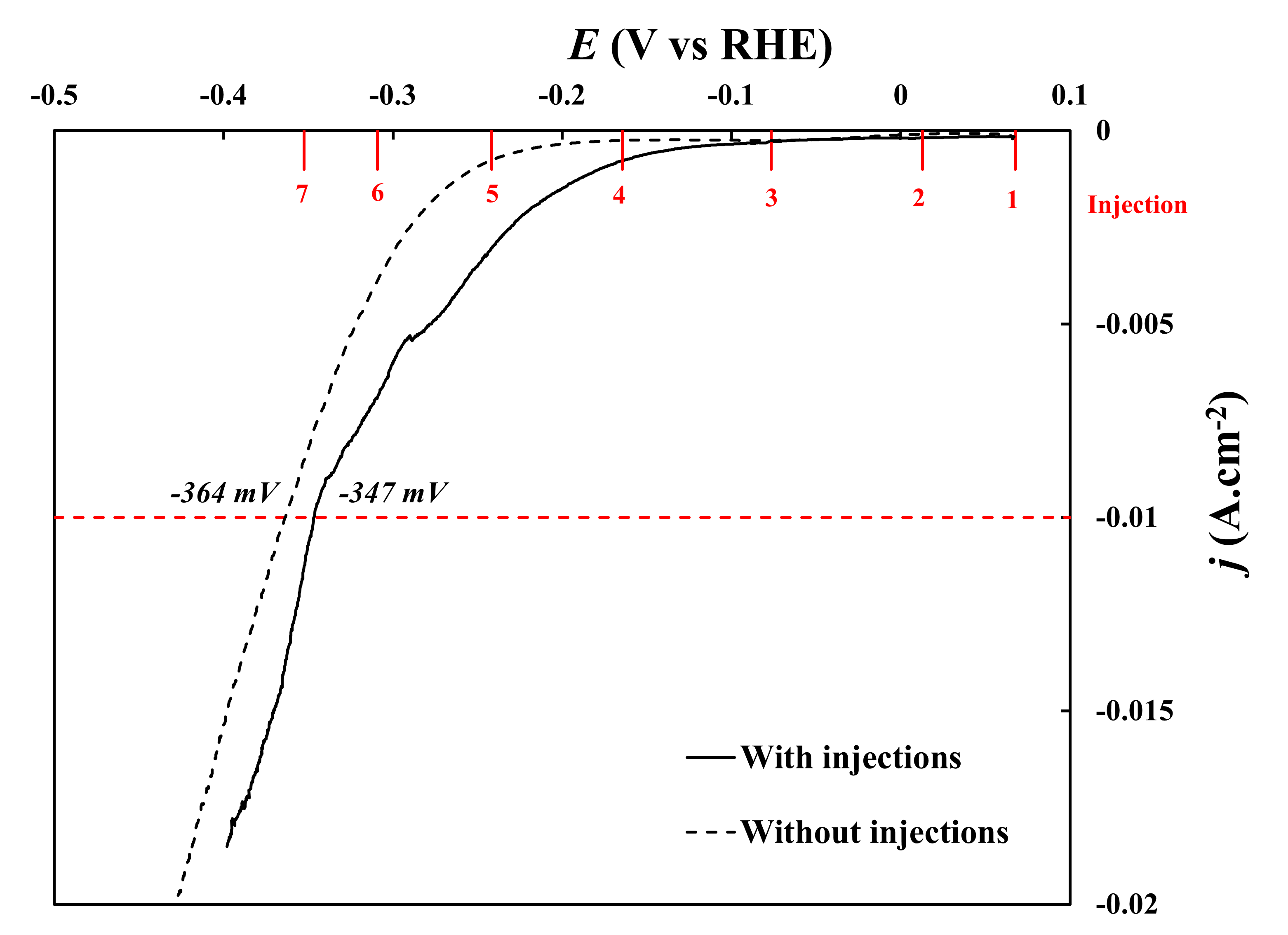


Figure A2 : LSV curves in HER region in 1.0 M KOH at 25°C and a scan of 0.3 mV.s^-1^ with and without H_2_O_2_ injection in silent conditions


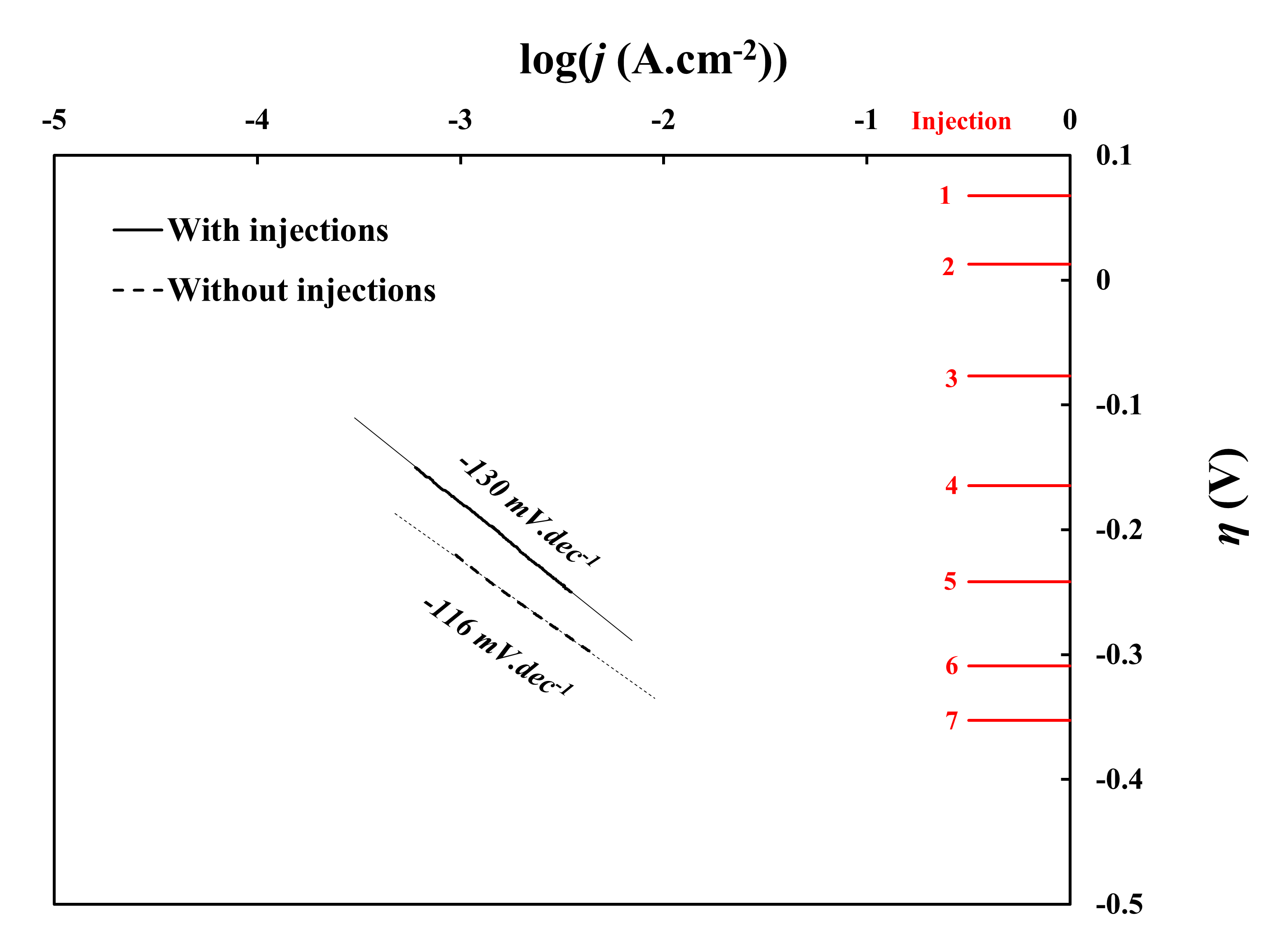


Figure A3 : Tafel slope in HER region in 1.0 M KOH at 25°C with and without H_2_O_2_ injection in silent conditions

According to Figures A2 and A3, the injection of H_2_O_2_ had no significant impact on the HER performances of the planar nickel electrode. The potential at -10 mA.cm^-2^ and the Tafel slope remained essentially unchanged, measured at -347 mV and -130 mV.dec^-1^_,_ respectively, compared to -364mV and -116 mV.dec^-1^ in the absence of injections.
